# Temporal dynamics of sensorimotor integration in speech perception and production: independent component analysis of EEG data

**DOI:** 10.3389/fpsyg.2014.00656

**Published:** 2014-07-10

**Authors:** David Jenson, Andrew L. Bowers, Ashley W. Harkrider, David Thornton, Megan Cuellar, Tim Saltuklaroglu

**Affiliations:** ^1^Department of Audiology and Speech Pathology, University of Tennessee Health Science CenterKnoxville, TN, USA; ^2^Department of Communication Disorders, University of ArkansasFayetteville, AR, USA; ^3^Speech-Language Pathology Program, College of Health Sciences, Midwestern UniversityChicago, IL, USA

**Keywords:** speech perception, speech production, EEG, mu rhythm, independent component analysis

## Abstract

Activity in anterior sensorimotor regions is found in speech production and some perception tasks. Yet, how sensorimotor integration supports these functions is unclear due to a lack of data examining the timing of activity from these regions. Beta (~20 Hz) and alpha (~10 Hz) spectral power within the EEG μ rhythm are considered indices of motor and somatosensory activity, respectively. In the current study, perception conditions required discrimination (same/different) of syllables pairs (/ba/ and /da/) in quiet and noisy conditions. Production conditions required covert and overt syllable productions and overt word production. Independent component analysis was performed on EEG data obtained during these conditions to (1) identify clusters of μ components common to all conditions and (2) examine real-time event-related spectral perturbations (ERSP) within alpha and beta bands. 17 and 15 out of 20 participants produced left and right μ-components, respectively, localized to precentral gyri. Discrimination conditions were characterized by significant (*p*FDR < 0.05) early alpha event-related synchronization (ERS) prior to and during stimulus presentation and later alpha event-related desynchronization (ERD) following stimulus offset. Beta ERD began early and gained strength across time. Differences were found between quiet and noisy discrimination conditions. Both overt syllable and word productions yielded similar alpha/beta ERD that began prior to production and was strongest during muscle activity. Findings during covert production were weaker than during overt production. One explanation for these findings is that μ-beta ERD indexes early predictive coding (e.g., internal modeling) and/or overt and covert attentional/motor processes. μ-alpha ERS may index inhibitory input to the premotor cortex from sensory regions prior to and during discrimination, while μ-alpha ERD may index sensory feedback during speech rehearsal and production.

## Introduction

It remains critical to disentangle the neural networks that allow an infinite array of co-articulated vocal tract gestures to be produced by a speaker and effortlessly sensed, recognized, and understood by a listener. Though these two complimentary and highly integrated processes often are examined independently, considerable recent effort has focused upon understanding how classical production mechanisms (e.g., the motor system) are involved in speech perception (D'Ausilio et al., 2012; Mottonen and Watkins, 2012; Murakami et al., 2013) and classical perception regions (i.e., auditory and somatosensory systems) are involved in production (Burnett et al., 1998; Stuart et al., 2002; Purcell and Munhall, 2006). Sensorimotor integration (SMI) provides an interface for speech perception and production and is fundamental to efficient verbal communication (e.g., Perrier et al., 1996; Rogalsky et al., 2011; Tourville and Guenther, 2011; Guenther and Vladusich, 2012; Moulin-Frier and Arbib, 2013). However, questions regarding the nature and timing of SMI prevail and relatively few studies address SMI in both speech perception and production within the same experiment (Wilson et al., 2004; Pickering and Garrod, 2007; Hickok et al., 2011; Adank, 2012).

Neuroimaging techniques have identified the auditory dorsal pathway (posterior temporal lobe, inferior parietal lobe, premotor cortex; PMC) as playing a role in both speech perception and production. In production, there is clear evidence of cooperation between feedforward and feedback systems for motor control (Houde and Nagarajan, 2011). Within speech perception, SMI is explained through independent yet convergent “dual streams” of neural activity (Scott and Johnsrude, 2003; Hickok and Poeppel, 2004; Hickok, 2009, 2012; Rauschecker, 2012; Specht, 2013). The ventral stream (predominantly within auditory regions) provides speech decoding and comprehension. The dorsal stream (including activity from sensorimotor regions) is thought to provide an audio-motor interface linking auditory to articulatory goals in speech perception. Though dorsal stream activity has been reported to be left-lateralized, there is recent evidence of bilateral organization (Cogan et al., 2014; Simmonds et al., 2014).

Despite evidence that the motor system is relatively inactive in non-degraded passive listening conditions (Scott et al., 2009; Szenkovits et al., 2012) and lesions demonstrating that damage to motor regions has little effect on the ability to perceive speech (Rogalsky et al., 2011), a number of perception tasks have been identified in imaging studies in which motor regions are recruited. These conditions typically have been those in which task demands are increased and include categorical discrimination of foreign phonemes (Callan et al., 2006), phoneme segmentation (Burton et al., 2000; Locasto et al., 2004; Burton and Small, 2006), and speech in noise (Osnes et al., 2011; Alho et al., 2012; D'Ausilio et al., 2012). Thus, motor system activity in speech perception may be context dependent, in addition to being variable across individuals (Szenkovits et al., 2012).

Given equivocal findings, the role of the motor system in speech perception is hotly debated. Perhaps a more pertinent question is the extent to which dorsal stream motor activity functionally enhances the perceptual process (Gallese et al., 2011). Hickok et al. (2011) maintain that contributions of the motor system are strictly modulatory and depend on the cognitive demands associated with a particular task. Others support a more functional role (Binder et al., 2004; Meister et al., 2007; D'Ausilio et al., 2009; Sato et al., 2009; Osnes et al., 2011; Grabski et al., 2013; Mottonen et al., 2013). In these studies, dorsal stream articulatory-motor based speech representations are associated with accurate speech perception in some tasks. However, to bolster understanding of the functional contributions of dorsal stream motor activity in speech perception, it is necessary to address the time-course of activity relative to acoustic stimulation in addition to task performance.

For example, Callan et al. (2010) used a combination of fMRI and magnetoencephalography (MEG), measuring PMC activity in a forced-choice, syllable discrimination in noise task. For correct discriminations, activity in the PMC preceded and immediately followed acoustic stimulation. These findings were interpreted as PMC activity functionally aiding in speech perception and were explained from a Constructivist perspective. That is, previous sensorimotor experiences bestow the motor system with the capacity to provide early top-down influences (in the form of predictive internal models) to help constrain sensory analysis and aid in perception (Sohoglu et al., 2012). In a manner that is also consistent with earlier analysis-by-synthesis theories (Stevens and Halle, 1967), these data suggest motor activity should be maintained while the internal model (i.e., hypothesis) is compared to the sensory consequences. Had motor activity in this study been found in a different time frame, other explanations might arise. For example, if motor activity only coincided directly with the occurrence of acoustic stimuli and was not related to functional performance, it might be interpreted from a Direct Realist viewpoint (Fowler, 1986), as a motor reflection of sensory stimulation. Similarly, motor activity that followed acoustic offset by 200 ms or more might be interpreted as covert rehearsal while the acoustic stimuli are kept in working memory (Callan et al., 2010).

Oscillatory models offer a time-sensitive means of examining neural processing of speech. These models posit a strong relation among phases of delta, theta, and gamma oscillations, and the temporal envelope of speech with respect to the encoding of discrete speech units (e.g., syllables). This relation reflects further evidence of auditory-motor coupling grounded in evolutionary adaptation for efficiency (Ghitza et al., 2012; Giraud and Poeppel, 2012). Measuring changes in spectral power across beta (15–25 Hz) and alpha (8–13 Hz) frequency bands may offer an additional method for understanding sensorimotor processing. Beta suppression is often associated with the anticipation of performance (Gladwin et al., 2006; Arnal, 2012; Bickel et al., 2012; Zaepffel et al., 2013) of motor activity and predictive (i.e., a priori) top-down coding for sensory analysis. Alpha bands dominate the human brain and the enhancement or suppression of alpha band power often is considered an indicator of cortical activation/inhibition (Klimesch, 2012). Event-related alpha desynchronization (ERD) is considered a release from inhibition for sensory gating and may also contribute to predictive coding. In addition, alpha power generally is suppressed with increased attentional and cognitive demands. Weisz and colleagues provide evidence of an independent auditory alpha generator, implicating a link to speech perception (Weisz et al., 2011; Obleser and Weisz, 2012). Additionally, in support of alpha sensitivity to speech perception, they found that magnitude of alpha suppression across a broad (prefrontal, temporal, parietal) network corresponded with reductions in speech stimulus intelligibility.

The rolandic mu (μ) rhythm is characterized by an arc-shape, alpha and beta band peaks, and typically localized to sensorimotor regions (Pineda, 2005; Hari, 2006). Spectral power within the μ-rhythm is often considered a down-stream measure of motor activity from the PMC (Pineda, 2005). Suppression of the power in the alpha band of the μ-rhythm (μ-alpha) has been used to measure sensorimotor activity in response to viewing biologically relevant (i.e., reproducible) vs. non-relevant visual stimuli such as hand (Oberman et al., 2005; Perry and Bentin, 2010) and face (Muthukumaraswamy and Johnson, 2004) movements, visually presented speech (Crawcour et al., 2009), and motor imagery tasks (Tamura et al., 2012; Holler et al., 2013). μ-alpha also suppresses to action-based sounds (Pineda et al., 2013), speech stimuli in segmentation tasks, and when identifying speech in noise (Cuellar et al., 2012). Additionally, Tamura et al. (2012) reported μ-alpha suppression to overt and imagined speech production under various types of auditory feedback. Their findings suggest that μ-alpha suppression in speech provides an index of feedback in audio-vocal monitoring. This interpretation seems logical considering that μ-alpha suppression is thought to arise from somatosensory activity when guidance is needed for ongoing movement (Hari, 2006). Considering also that μ-beta suppression is indicative of motor activity, identifying patterns of μ-alpha and μ-beta ERS/ERD across speech tasks is likely to reveal further important information about the timing of motor and sensory contributions to SMI in speech processing.

To this end, Bowers et al. (2013) recently employed an EEG technique to study SMI during speech perception, adapting a similar design from an fMRI and MEG study (Callan et al., 2010). Specifically, participants passively listened to and actively discriminated (i.e., forced choice, same or different) between pairs of syllables (/ba/ and /da/) and tone sweeps presented in different signal-to-noise ratios (SNRs). Raw data from 30 EEG recording channels were analyzed via independent component analysis (ICA). ICA is blind-source separation (i.e., linear decomposition) tool that can be used both as a strong filter and a means of independent and spatially fixed sources of neural activity (Delorme and Makeig, 2004; Makeig et al., 2004; Onton et al., 2006). Left and right μ-rhythm component clusters with characteristic spectral peaks at ~10 Hz and ~20 Hz (Hari, 2006), maximally localized to the sensorimotor cortex with activation extending into the PMC, were identified in most participants. Time-frequency analysis of μ components using event-related spectral perturbation (ERSP) analysis showed ERD in the beta band that was strongest when speech was accurately (>95% correct) discriminated in noise with a SNR of + 4 dB. Most importantly, in this condition only, μ-beta suppression (i.e., motor activity) began prior to speech perception and peaked immediately following stimulus offset. The findings were interpreted in accord with Callan et al. (2010) and others, suggesting that PMC/sensorimotor regions can readily contribute to speech perception (e.g., Skipper et al., 2007). From an oscillatory perspective, they were interpreted as evidence of early top-down influences from the motor system (i.e., internal models), helping to constrain auditory analysis in shared channels between sensorimotor and auditory regions (Arnal and Giraud, 2012).

Bowers et al. (2013) demonstrated that this event-related EEG technique with subsequent ICA/ERSP analysis is suitable for measuring SMI in speech perception. However, it is important to note that all their conditions employed background noise. Mottonen et al. (2013) used rTMS to impair motor representations from the lips and found impaired speech discrimination, suggesting that auditory-motor dorsal stream activity is important for speech discrimination in normal as well as degraded conditions. Alho et al. (2012) reported similar findings using evoked potentials. More generally, μ suppression has been found in anticipation of correctly predicted visual targets suggesting functional support in the task from attentional/motor networks (Bidet-Caulet et al., 2012). Hence, a salient question that remains pertains to the extent to which patterns of beta suppression in noisy speech discrimination tasks can disassociate the influences of a degraded listening environment from those functionally related to accurate categorical perception (Specht, 2014). To further understand how μ rhythms respond in speech discrimination tasks, it is necessary to examine the time-course of μ ERS/ERD in a quiet discrimination condition.

Regions within the dorsal stream help mediate sensorimotor control in speech production (Houde and Nagarajan, 2011; Hickok, 2012; Rauschecker, 2012). Feed-forward motor plans are generated that, once properly trained, allow for fluid generation of co-articulated speech gestures at an appropriate speaking rate (Tourville and Guenther, 2011). In addition, inverse forward models (i.e., efference copies) of predicted sensory consequences are sent from motor regions (i.e., premotor/motor cortex) to higher order auditory (e.g., superior temporal sulcus) and somatosensory (e.g., inferior parietal lobe) sites for dynamic comparisons with auditory and somatosensory production, providing the neurophysiological basis for an ongoing feedback loop. As forward prediction is compared with the intended targets and subsequently integrated with the true sensory (i.e., acoustic) and somatosensory consequences of production, corrective feedback is returned to motor control centers in a manner so efficient as to allow for online correction should a mismatch occur between predicted consequence and the articulatory goal. According to dynamic state feedback control (SFC) models, across the time course of any given speech production, complex dynamic interplay can exist between feedforward and feedback control in response to ongoing changes in vocal tract configurations and acoustic/somatosensory perturbations (Ventura et al., 2009; Houde and Nagarajan, 2011; Golfinopoulos et al., 2011; Hickok, 2012, 2014). Hence, as in speech perception, the addition of temporal data from regions within dorsal stream networks is likely to help foster a better understanding of the feedforward and feedback dynamics in speech production.

The largest obstacle to deploying imaging techniques with high temporal resolution such as EEG and MEG to speech production is signal contamination from muscle artifact. It is well known that myogenic activity from eyes (e.g., blinking), lips, head, and jaw produces robust electrical activity in frequency ranges broad enough to spuriously influence most neural activity. In addition, due to volume conduction, the effects of myogenic activity are not focal and can influence recordings from all cranial electrodes (McMenamin et al., 2011). Due to this limitation, EEG and MEG studies targeting language production networks have employed a variety of experimental designs intended to circumvent overt speech production. These designs have typically involved delayed or covert speech production. As evidence exists showing similarities in neural activity in overt and covert production tasks, Tian and Poeppel (2010, 2012) including the generation of internal models (Sams et al., 2005; Tian and Poeppel, 2010), covert production often provides a viable substitute for overt production tasks. However, in terms of SMI, the two tasks are different and may not share all the same neurophysiology (Ganushchak et al., 2011), especially in some pathological conditions with compromised sensorimotor control such as stuttering (Max et al., 2003; Loucks and De Nil, 2006; Watkins et al., 2008; Hickok et al., 2011; Cai et al., 2014; Connally et al., 2014). Other studies have stopped short of measuring activity during production, instead relying on oscillatory data from a time window prior to actual production. In this vein, a number of ERP studies have measured ‘lexical’ access and morphological encoding strategies (Hirschfeld et al., 2008; Costa et al., 2009; Dell'acqua et al., 2010; Strijkers et al., 2010). Whole head MEG data have revealed patterns of μ-alpha suppression in auditory regions with μ-beta suppression in auditory-motor (i.e., dorsal stream) integration regions (Gehrig et al., 2012). Similarly, Herman et al. (2013) measured real-time changes in oscillatory data from syllable encoding and pre-production time periods to identify discrete input/output operations within the dorsal stream phonological loop, again highlighting the value of temporal information.

Improvements in source estimations and data analysis techniques, along with continued widespread availability appear to be contributing to a resurgence of EEG. ICA has been suggested as an effective technique for separating neural from myogenic activity on the basis of the assumption of temporal and spatial independence of components. Therefore, especially when stereotypical in nature, myogenic activity can be separated from neural activity in the unmixing process following ICA training on sufficient data (Delorme and Makeig, 2004; Onton et al., 2006; Gwin et al., 2010). The use of ICA in this capacity has been demonstrated to remove movement artifact while performing hand movements (Shou et al., 2012), walking and running (Gwin et al., 2010; Lau et al., 2014), and in distinguishing distinct patterns of electro-cortical activity in knee vs. ankle movements (Gwin and Ferris, 2011). However, its application to speech production has been limited. Though seemingly daunting, Tran et al. (2004) reported successfully using ICA to remove artifact from stuttered speech in children. In addition, other studies have demonstrated that ICA can be used to reveal neural activity not evident in univariate analyses (Geranmayeh et al., 2012; Simmonds et al., 2014).

Though there is reason for optimism regarding the potential use of EEG with ICA for measuring neural activity in speech production tasks (Ganushchak et al., 2011), concerns remain regarding the potential for ICA to adequately remove all muscle artifact. These include reduced validity for localization in intracerebral space, the fact that muscle artifact is often non-stereotypical and therefore not always suited for identification via ICA, and that a substantial portion of the variance in the whole EEG signal (i.e., up to 67% of components) can be accounted for by pure myogenic activity, reducing spectral power in neural components of interest (McMenamin et al., 2009; Shackman et al., 2009; McMenamin et al., 2010, 2011). It is clear that preliminary investigations using ICA in speech production should proceed cautiously using simple productions.

As a launching point, the current study focuses on activity from μ components for the following reasons. First, they are ubiquitously found in EEG recordings, particularly when identified via ICA. Thus, the possibility of yielding μ components in ICA decomposition remains high even when muscle components predominate. Second, μ rhythms typically are localized to primary motor/PMC regions, which are key sites within the dorsal stream. The PMC, in particular, is bi-directionally connected to higher-level auditory and somatosensory regions via the arcuate and longitudinal fasciculi. Its location and connectivity allow it to serve as an important intermediary for integrating forward prediction (internal modeling) and sensory feedback in both perception and production (Houde and Nagarajan, 2011; Rauschecker, 2012). Third, μ-ERS/ERD already has revealed real-time data interpreted as predictive coding in speech perception (Bowers et al., 2013). Alpha and slow beta bands which are contained within the μ rhythm are the only frequency domains that display ERS/ERD sensitivity to stimulus and/or task (Klimesch, 2012). Therefore, further time-frequency analyses of μ rhythms potentially may reveal important information about SMI in production.

There are two main goals in this study. The first is to bolster understanding of the timing and function of dorsal stream activity in speech perception by examining ERS/ERD patterns in quiet and noisy discrimination conditions. The second is to provide initial evidence that, via the application of ICA/ERSP, the use of EEG can be extended into the realm of speech production. Collectively, the intention is to show that ICA can be used accurately to identify dorsal stream sensorimotor μ components common to both speech perception and production. It is first hypothesized that right and left μ components, localized to sensorimotor/PMC regions, will be found across perception and production conditions. By placing EMG electrodes on the upper and lower lip, it also is anticipated that ICA will identify prominent perilabial muscular activity. Once μ components are identified, the second hypothesis is that ERSP analyses will provide differential time-frequency measures of alpha and beta ERS/ERD. Real-time oscillatory changes in the spectral power of alpha and beta bands of the μ rhythm are expected to provide novel information regarding the timing of SMI in speech perception and production that may be interpreted via dual stream/SFC models. Additionally, significant activity from perilabial components is expected only in overt production, allowing it to be mapped in real-time to SMI activity.

## Materials and methods

### Participants

Twenty right-handed English-speaking adults (17 female and 3 males) with a mean age of 23.94 years (range 21–39 years) were recruited from audiology and speech pathology classes at the University of Tennessee Health Science Center. Participants reported no diagnosed history of communicative, cognitive, or attentional disorders. Handedness dominance was assessed using the Edinburgh Handedness Inventory (Oldfield, 1971). This study was approved by the Institutional Review Board of the University of Tennessee Health Science Center. Prior to the experiment, participants provided signed informed consent on a document approved by the Institutional Review Board.

### Stimuli

#### Perception

**Figure 1:**
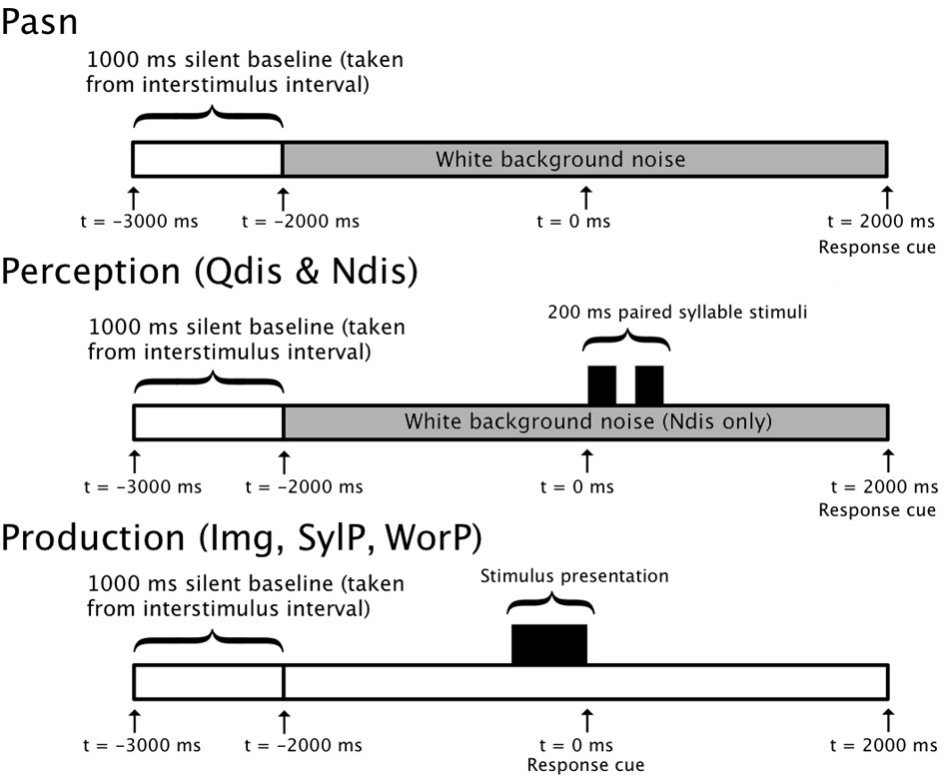
**Single-trial timelines for perception and production conditions**. Timelines for 5000 ms (−3000 to 2000 ms) in passive noise condition (Pasn), discrimination conditions (Qdis & Ndis), and production conditions (Img, SylP, and WorP).

/ba/ and /da/ syllables were created using AT&T naturally speaking text-to-speech software which employs synthetic analogs of a human male speaker. Syllable pairs were generated such that half of the stimuli were composed of different syllables (e.g., /ba/ and /da/) and the other half were identical (e.g., /ba/ and /ba/). The stimuli were low-pass filtered below 5 KHz and normalized for root-mean-square (RMS) amplitude. Each syllable was 200 ms in duration. Each syllable pair was also separated by 200 ms, resulting in a total of 600 ms from the first syllable onset to the second syllable offset (Figure 1).

For one condition (discrimination in noise; Ndis), syllable pairs to be discriminated were embedded in white noise with a SNR of +4 dB. This SNR was chosen as it has been shown previously (Bowers et al., 2013) to produce discrimination accuracies > 95% in a similar group of participants. In the other discrimination condition (discrimination in quiet; Qdis), syllable pairs were presented without background noise. To prevent discrimination response bias (Venezia et al., 2012), in both Qdis and Ndis stimuli sets, there were equal numbers of syllable pairs that were identical as there were different.

#### Production

Speech targets were syllable pairs, similar to those used in the discrimination tasks above, and tri-syllable nouns (initiated with /b/ or /d/ and followed by a vowel). They were displayed centered in Microsoft PowerPoint slides with plain black backgrounds in large white Arial font (size 56). Figure 1 shows the timelines for epochs in both perception and production conditions.

### Design

A 6-condition within-subject design was employed. Based on extant literature, the conditions were created to increase motoric demands incrementally (i.e., from the perceiving of white noise to overtly producing tri-syllable words). Participants were required to:

1. passively listen to white noise (Pasn).
2. discriminate (same or different) between pairs of syllables in a quiet background (Qdis).
3. discriminate (same or different) between pairs of syllables in a noisy background (Ndis).
4. imagine producing a pair of syllables (Img).
5. overtly produce (i.e., say) a pair of syllables (SylP).
6. overtly produce (i.e., say) tri-syllable nouns initiated by /b/ or /d/ and followed by a vowel (WorP).

Thus, condition 1 (Pasn) required no discrimination and was a control task for the two discrimination conditions (Qdis and Ndis). Conditions 2–5 employed /ba/ and /da/ syllables. Conditions 2 and 3 (Qdis and Ndis) required same/different discriminations of random /ba/ and /da/ combinations, while conditions 4 and 5 required covert (Img) and overt (SylP) production of randomly selected /ba/ and /da/ combinations. Condition 6 (WorP) also required overt production, but in this condition tri-syllable nouns were used as opposed to the 2-syllable combinations employed in the SylP condition. In the WorP condition, words meeting these criteria were selected from Blockcolsky et al. (2008). Examples of these words include “dialog,” “butterscotch,” “daffodil,” and “buffalo.”

### Procedure

The experiment was conducted in an electronically and magnetically shielded, double-walled, sound-treated booth. Participants sat in a comfortable reclining armchair with their heads and necks well supported. Compumedics NeuroScan Stim 2 version 4.3.3 software was used to present stimuli to participants via a PC computer and record button-press responses. A button-press response was required for all three perception conditions because anticipation of a button-press has previously been known to elicit μ-rhythm ERD (Makeig et al., 2004; Graimann and Pfurtscheller, 2006; Hari, 2006). Hence, in the Pasn condition, the button-press was used as a control for the required button-press response in the discrimination conditions and to ensure that participants were paying attention in each trial. The cue to respond was a 100 ms, 1000 Hz tone that was presented at the end of the epoch (i.e., +2000 ms). In the Pasn condition, participants were instructed simply to listen passively to the noise and press the designated button after hearing a pure tone cue in each trial. Designation of button-press responses (right or left hand) was counterbalanced across all subjects and experimental conditions. Performance in the discrimination conditions was evaluated by calculating the percentage of correct trials.

In the production conditions, stimuli appeared on a 69.5 × 39.0 cm display placed 132 cm in front of the reclining chair. The stimuli appeared on the screen for 1 s. Participants were instructed to begin their production response immediately when the stimulus disappeared from the monitor. In the Img condition participants were told to imagine saying (i.e., covertly producing) the pair of syllables while refraining from making any overt articulatory movements or vocalization. In the SylP and WorP condition, participants were instructed to speak the syllable pair or word in their normal speaking voice. All overt speech productions were easily completed in the time window (2 s) following the cue to speak. All conditions were presented in two blocks of 40 trials each. The order of the 12 blocks (6 conditions × 2 blocks) was randomized for each participant.

### EEG acquisition

Sixty-eight electrode channels were used to acquire whole-head EEG data. These included two electromyography (EMG) and two electrocardiogram (ECG) electrodes. Electrode configuration was based upon the extended international standard 10–20 (Jasper, 1958) method using an unlinked, sintered NeuroScan Quik Cap (Towle et al., 1993). All recording electrodes were referenced to the common linked left (M1) and right (M2) mastoids. The electro-oculogram (EOG) was recorded by placing electrodes on the left superior orbit and the left inferior orbit (VEOG) as well as the lateral and medial canthi of the left eye (HEOG) to monitor vertical and horizontal eye movements, respectively. The two surface electromyography (EMG) electrodes were placed at midline above the upper lip and below the lower lip for the purposes of collecting perilabial EMG data related to speech production.

EEG data were collected using Compumedics NeuroScan Scan 4.3.3 software and the Synamps 2 system. The raw EEG data were filtered (0.15–100 Hz) and digitized via a 24-bit analog-to-digital converter at a sampling rate of 500 Hz. Data collection was time-locked to time point zero at the onset of acoustic stimuli delivery in speech perception trials and the cue to begin speaking in production trials. Thus, in the perception conditions, time zero referenced the acoustic onset of the first syllable. In the production conditions, syllable and word stimuli were orthographically displayed on the monitor between times -1000 ms and zero. Hence, disappearance of the text at time zero was the cue for participants to begin speaking (Figure 1).

### EEG data processing

EEGLAB 12 open source software (Delorme and Makeig, 2004) was used to process all EEG data by performing the following steps for individual and group processing/analysis.

1. Individual processing/analysis:
  - 12 raw EEG files (6 conditions x 2 blocks) were pre-processed for each participant.
  - Independent component analysis (ICA) was performed on all concatenated files across all conditions for each participant.
  - All neural and non-neural dipoles were localized for each independent component (IC) identified.
2. Group analysis:
  - Using the STUDY module of EEGLAB 12, two separate analyses were performed using ‘in head’ only (neural) and ‘all’ (neural and non-neural) ICs.
  - Principal component analysis (PCA) subsequently was used to identify and cluster common components across participants.
  - Left and right μ clusters were identified from the ‘in-head’ STUDY, whereas the EMG cluster representing perilabial muscle activity was identified from the ‘all’ STUDY.
  - μ clusters were localized using equivalent current dipole (ECD) and current source density (CSD) analyses.
  - Time-frequency analyses (measuring changes in spectral power across time) were performed by measuring event-related spectral perturbations (ERSP) in the left and right μ clusters as well as in the EMG cluster.

Details of each step in the data processing / analyses are described below.

#### Processing/analysis of EEG data from each participant

**Figure 2:**
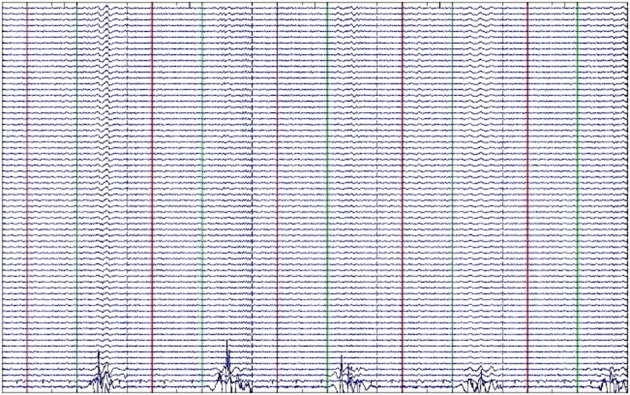
**Stereotypic muscle activity from perilabial EMG electrodes**. Example of filtered and epoched EEG data from the WorP condition showing stereotypical EMG activity during speech production.

***Data pre-processing***. Raw data from both 40-trial blocks in each condition were: (1) appended to make a single 80 trial data set for each condition; (2) downsampled to 256 Hz to decrease computational requirements for ICA processing; (3) epoched into 5000 ms segments with individual epochs spanning from −3000 to +2000 ms around time zero; (4) band-pass filtered (3–34 Hz) to ensure that alpha and beta could be identified while filtering muscle movement from surrounding frequencies; (5) re-referenced to mastoid electrodes; (6) visually inspected for gross artifact (> 200 μV), which was manually removed; and (7) pruned to remove trials with incorrect responses or response latencies greater than 2 s in the Qdis and Ndis conditions, although few trials were removed (see below). A minimum contribution of 40 epochs per participant per condition was required for inclusion in the experiment. However, the average number of usable trials across participants per condition far exceeded the minimum of 40 required for inclusion. Figure 2 shows an example of EEG activity from 5 trials in one participant in the WorP condition following filtering and epoching. Critically, the muscle activity from the EMG component appears to be relatively stereotypical in nature (e.g., Figure 2), thereby facilitating ICA efforts to separate the neural activity from the muscle activity in the subsequent ICA signal decomposition.

***Independent component analysis (ICA)***. Prior to ICA training, pre-processed EEG data for each participant were concatenated across all 6 conditions so that a single set of ICA weights could be obtained. This allowed for a comparison of activity to be made across conditons within spatially fixed ICs. An extended Infomax algorithm (Lee et al., 1999) was used to decorrelate the data matrix prior to ICA rotation. ICA training was provided using the “extended *runica*” algorithm in EEGLAB 12 with an initial learning rate set to 0.001 and a stopping weight of 10–7. Following decomposition, 66 ICs were yielded for each participant reflecting the total number of recording electrodes (68 – 2 reference electrodes, M1 and M2). Scalp maps for each IC were obtained by projecting the inverse weight matrix (W^−1^) back onto the spatial EEG channel configuration.

Following ICA decomposition, equivalent current dipole (ECD) models for each component were computed using a boundary element model (BEM) in the DIPFIT toolbox, freely available at sccn.ucsd.edu/eeglab/dipfit.html (Oostenveld and Oostendorp, 2002). Standard 10–20 electrode coordinates were warped to the head model followed by automated coarse-fitting to a BEM, yielding a single dipole model for each of 1320 ICs (66 ICs × 20 participants). Dipole localization requires back-projecting the signal to a source that may have generated the scalp potential distribution for a given IC, and then computing the best forward model to explain the highest percentage of scalp map variance (Delorme et al., 2012). Residual variance (RV) in dipole localizations were also computed, referring to the potential mismatch between the initial scalp map and the forward projection of the ECD model.

#### Group data analyses

***EEGLAB STUDYs***. Group data analyses were conducted via the EEGLAB STUDY module. The STUDY module allows ICA data from multiple participants across conditions to be analyzed using specified designs. In the current study, the designs specified were dictated by the within-subjects conditional differences of interest. The STUDY module allows further filtering to be applied with respect to the RV in dipole localization and inclusion vs. exclusion of out-of head dipoles. Thus, ICA files with dipole information from each individual (see above) were applied to the two separate STUDY modules. For the purposes of measuring neural activity, only “in-head” dipoles with RV< 20% were analyzed.

For the purposes of identifying perilabial EMG activity, a second STUDY was conducted that included “all” dipoles from in head and outside the head. In this second STUDY, the RV criterion was raised to 50% (Gramann et al., 2010) dipoles because EMG activity emanates from outside the head and by nature, muscular movement incurs higher unexplained RV.

***Principal component clustering of ICs***. In both the “in head” and “all” STUDYs, component pre-clustering was performed on the basis of common scalp maps, dipoles, and spectra. The K-means statistical toolbox (implemented in EEGLAB; Delorme and Makeig, 2004) then used these criteria to group similar components from each participant via PCA. After removal of outliers (3 SD from any cluster mean), components from the “in head” STUDY were assigned to 20 possible neural clusters, which included left and right sensorimotor μ clusters. Components in the “all” STUDY were assigned to 66 possible clusters and included one non-neural cluster depicting perilabial EMG activity.

Final component designation to left and right μ clusters was based primarily on the PCA followed by individual inspection of spectra, scalp maps, and dipoles of all components within those clusters and neighboring clusters. Final inclusion criteria for membership to μ clusters included localization to BA 1–4, and 6 (i.e., somatosensory regions, primary motor and premotor regions) and characteristic μ spectra, though over 90% of components emanated from BA 6.

Components in the “all” STUDY were assigned to 66 possible clusters, most of which, as expected, depicted non-neural activity. The cluster characterizing perilabial EMG activity was found on the basis of dipole location and ERSP analysis showing activity only in overt production tasks (see below).

***μ cluster source localization***. ECD source localization is simply from the average (x, y, z) coordinate of all the IC dipoles (identified via the DIPFIT module) within a given cluster. Alternatively, standardized low-resolution brain electromagnetic tomography (sLORETA) uses current source density (CSD) distribution from electrical potential measures across the scalp to address the inverse problem and provide an estimate of source localization (Pascual-Marqui, 2002). The head model uses a Talairach cortical probability brain atlas, digitized at the Montreal Neurological Institute (MNI). EEG electrode locations are cross-registered between spherical and realistic head geometry (Towle et al., 1993). Spatial resolution of 5 mm is achieved by sampling 6239 voxels in 3-D brain space. For each IC that contributed to the two μ clusters, the inverse weight projections on the original EEG channels were exported to the sLORETA. Cross-spectra were computed and mapped to the standard Taliarach brain atlas cross-registered with the Montreal Neurological Institute (MNI) coordinates, yielding sLORETA estimates of CSD for left and right μ dipoles in the “in-head” STUDY. To evaluate the statistical significance of dipole locations across participants, statistical comparisons relative to zero (i.e., no activation) were computed (Grin-Yatsenko et al., 2010). Paired (Student) *t*-tests were conducted on frequencies between 4 and 33 Hz (1000 frames) with the smoothing parameter set to 1 (single common variance for all variables), using 5000 random permutations yielding corrected *t*-value thresholds and statistical significance (p < 0.001) for all 6239 voxels.

While these two methods of EEG source localization were expected to produce similar results (Bowers et al., 2013), for reliability purposes it was deemed useful to use both techniques.

***Time-frequency analysis (change in spectral power across time)***. ERSP analyses were used to compute changes (scaled in normalized dB units) in power across time (i.e., time-frequency analysis) within the spectral range of interest (4–33 Hz). Time-frequency transforms were derived using a Morlet sinusoidal wavelet set at 3 cycles at 3 Hz, rising linearly to 20 cycles at 40 Hz. The 1000 ms pre-stimulus period was selected from the silent inter-trial interval to serve as a baseline for each trial. These baselines were constructed from a surrogate distribution based on estimates of spectral power from 200 randomly selected latency windows from within the 1000 ms inter-trial interval (Makeig et al., 2004). Subsequent individual ERSP changes from baseline over time were computed using a bootstrap resampling method (*p* < 0.05 uncorrected). The single trial current for all experimental conditions for frequencies between 7 and 30 Hz and times from −500 to 1500 ms were entered in the time-frequency analyses.

In the “in-head” STUDY, differences in cross-conditional ERSPs in right and left μ clusters were computed using permutation statistics (2000 permutations) with a 95% confidence interval (*p* < 0.05). The random distribution represents the null hypothesis that no condition differences exist. Type I error was controlled by correcting conservatively for false discovery rates (*p*FDR; Benjamini and Hochberg, 2000). Statistical analysis in the perception conditions used a 1 × 3 (Pasn, Qdis, Ndis) repeated measures ANOVA design. *Post-hoc* comparisons examined differences between Pasn vs. Qdis and Pasn vs. Ndis conditions. In the production conditions, a 1 × 3 repeated measure ANOVA design examined differences in ERSP activity across the Img, SylP, and WorP conditions. A *post-hoc* paired comparison examined differences between SylP and WorP conditions. In the “all” STUDY, cross-conditional ERSPs were computed in the production conditions using a 1 × 3 repeated measure ANOVA design.

## Results

### Discrimination accuracy

In participants that contributed to μ clusters, the average number of useable trials (out of 80) across participants in each condition were: Pasn = 73.8 (*SD* = 7.2); Qdis = 74.8 (*SD* = 4.6); Ndis = 69.0 (*SD* = 11.4); Img = 75.0 (*SD* = 5.8); SylP = 71.1 (*SD* = 7.4); WorP = 69.9 (*SD* = 8.0). In the Qdis condition, all participants discriminated with 91–100% accuracy. In the Ndis condition, all except one participant discriminated with 84–100% accuracy. The remaining participant discriminated with 65% accuracy. The average discrimination accuracies in the Qdis and Ndis conditions were 97.3 and 94.4%, respectively. A paired *t*-test indicated that mean discrimination performance was not significantly different (*p* > 0.05) in these conditions. The average response latencies in the Qdis and Ndis conditions were 504 and 545 ms, respectively. A paired *t*-test again indicated that these latencies were not significantly different (*p* > 0.05). Together, these findings suggest that both discrimination tasks were performed with similar high levels of accuracy and efficiency. It should be noted again that trials with incorrect discriminations were eliminated from the data so the EEG analysis was limited to correct productions only.

### μ and EMG cluster characteristics

**Figure 3:**
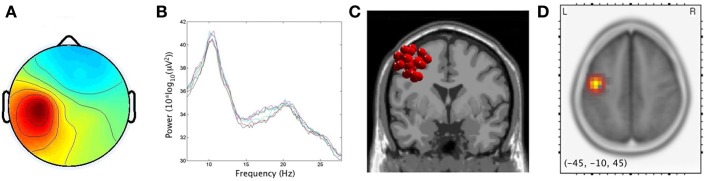
**Cluster results for left μ component. (A)** mean scalp potential distribution (W^−1^) scaled to RMS microvolts, **(B)** mean spectra of the components within the cluster for each condition, **(C)** distribution of equivalent current dipoles within the cluster, and **(D)** maximum current source density voxels (***t***-values) with greater values in darker colors and smaller values in lighter colors (at *p* < 0.001 corrected for multiple comparisons).

**Figure 4:**
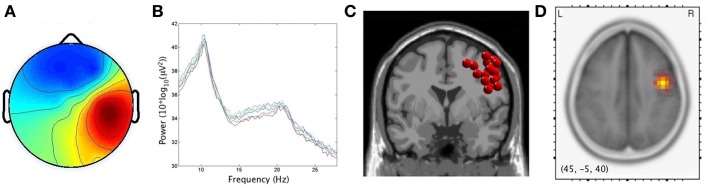
**Cluster results for right μ component. (A)** mean scalp potential distribution (W^−1^) scaled to RMS microvolts, **(B)** mean spectra of the components within the cluster for each condition, **(C)** distribution of equivalent current dipoles within the cluster, and **(D)** maximum current source density voxels (***t***-values) with greater values in darker colors and smaller values in lighter colors (at *p* < 0.001 corrected for multiple comparisons).

As predicted by the first hypothesis, 17/20 and 15/20 participants produced components with< 20% unexplained RV that contributed to left and right μ clusters, respectively. For the left μ cluster, the average Talaraich ECD location was [−41, 4, 46], while on the right it was [46, 0, 39]. The percentage of unexplained RV in these single dipole models was 10.1 and 8.7% for the left and right hemispheres, respectively. sLORETA analyses revealed significantly activated voxels (*p* < 0.001) associated with μ clusters. Maximum current source densities were found at Talairach [−45, −10, 45] on the left vs. [45, −5, 40] on the right. In accord with findings by Bowers et al. (2013), the two localization techniques produced similar results, here allowing sources of μ activity to be maximally localized within the precentral gyri with activity spreading across the PMC and sensorimotor regions. Figures 3 and 4 respectively display the scalp maps (A), spectra (B), ECD dipole clusters (C) and CSD maxima (D) for left and right μ clusters, respectively. The EMG cluster was characterized by non-neural ICs with an average of 21.3% unexplained RV.

### Time-frequency analyses in perception (Pasn, Qdis and, Ndis) conditions

**Figure 5:**
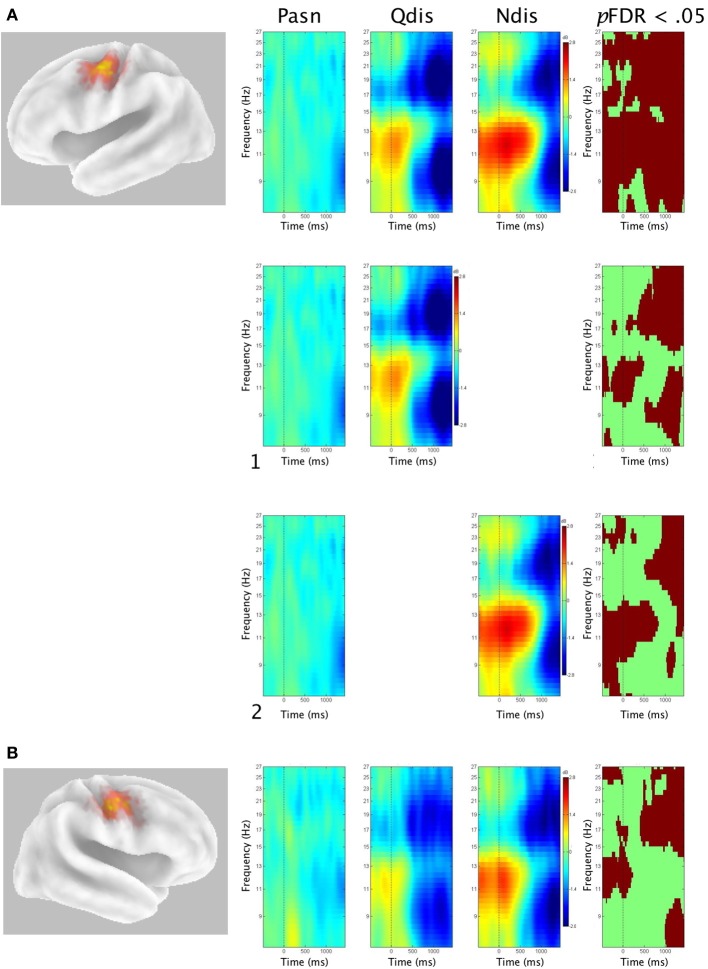
**Mean left and right ERSPs and sLORETA solutions for perception conditions**. Rows A and B show sLORETA solutions for left and right μ clusters, respectively, depicted on a 3D Van Essen average template, followed by mean time-frequency ERSPs (event-related spectral perturbations) as a function of perception conditions, before, during, and after stimulus offset for **(A)** left μ clusters with (1) contrasts between Pasn and Qdis and (2) contrasts between Pasn and Ndis; and **(B)** right μ clusters (red, ERS, blue, ERD). The last frame in each row shows significant differences across conditions (*p*FDR< 0.05).

Figure 5 shows Van Essen maps (generated using sLORETA) of significant voxels contributing to left (A) and right (B) μ clusters, followed by time-frequency (ERSP) analyses within the 7–30 Hz bandwidth. The ERSP analyses show significant ERS/ERD changes from baseline in the Pasn, Qdis, and Ndis conditions. The last frame in each row shows statistical ERSP differences across conditions (*p*FDR < 0.05), thus supporting the second hypothesis.

For the left μ cluster, relative to the Pasn, alpha ERS began prior to acoustic stimulation and gradually gave way to alpha ERD beginning in low alpha frequencies (8–11 Hz) following acoustic offset in both discrimination conditions (Qdis and Ndis). Beta ERD in both discrimination conditions began in a narrow bandwidth (17–19 Hz), growing stronger and spreading across beta frequencies during and immediately following the acoustic stimulation condition. *Post-hoc* analyses (shown in Figures 5A1,A2) show differential patterns of significant beta ERD and alpha ERS/ERD in Pasn vs. Qdis comparisons and Pasn vs. Ndis comparisons.

Patterns of alpha/beta ERS/ERD were similar yet weaker and more diffuse in the right μ cluster compared to those on the left. It followed that *post-hoc* ERSP comparisons of Qdis and Ndis to Pasn comparisons for right μ activity did not yield additional data of interest.

### Time-frequency analyses in production (Img, SylP and, WorP) conditions

**Figure 6:**
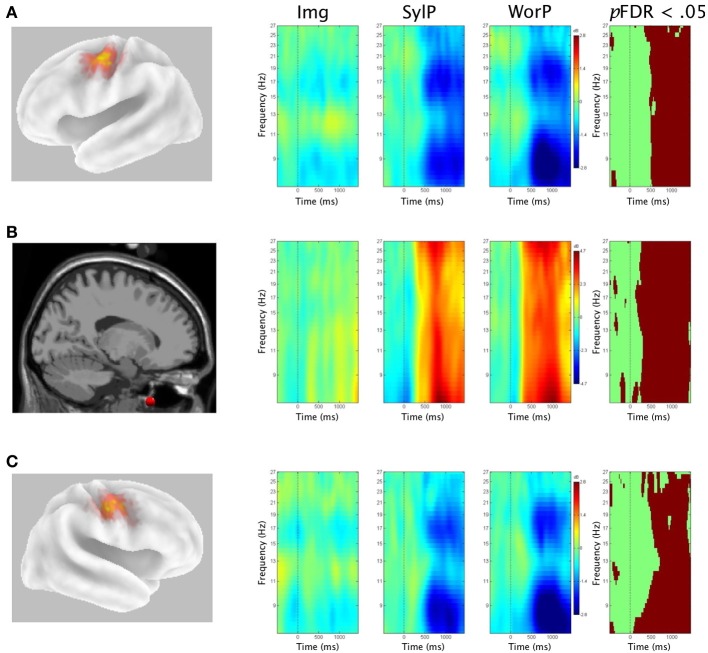
**Mean left and right ERSPs and sLORETA solutions for production conditions**. Rows A and C show sLORETA solutions for left and right μ clusters, respectively, depicted on a 3D Van Essen average template, followed by mean time-frequency ERSPs (event-related spectral perturbations) as a function of production conditions, before, during, and after stimulus offset. The last frame in each row shows significant differences across conditions (*p*FDR < 0.05). Row B shows activity within the EMG component from perilabial myogenic activity.

Figure 6 shows Van Essen maps (generated using sLORETA) of significant voxels contributing to left (A) and right (C) μ clusters, followed by time-frequency (ERSP) analyses within the 7–30 Hz bandwidth. The ERSP analyses show significant ERS/ERD changes from baseline in the Pasn, Qdis, and Ndis conditions. The last frame in each row shows statistical ERSP differences across conditions (*p*FDR < 0.05), again supporting the second hypothesis. Figure 6B shows the average ECD dipole location for the EMG components followed by ERSP analyses with statistical differences across conditions.

Significant EMG ERS (i.e., activity indicative of lip movement) in the SylP and WorP conditions began ~300 ms after the cue to initiate speech. In both left and right μ clusters, alpha/beta ERD relative to baseline began in all production conditions up to 500 ms before the cue to speak. However, alpha/beta ERD in SylP and WorP conditions was significantly stronger (*p*FDR < 0.05) than in the Img condition during overt speech production (i.e., coinciding with EMG activity). *Post-hoc* analyses in both left and right μ clusters showed no ERSP differences in SylP vs. WorP conditions.

As μ-ERD was significantly weaker in Img relative to overt production (SylP and WorP) conditions, ERSPs for all components contributing to left and right μ clusters were examined in the Img condition. On the left, only 8 of 17 participants displayed μ-ERD in this condition. The others either showed ERS or negligible change. On the right, 6 of 15 showed μ-ERD, 2 showed patterns of alpha ERS with beta ERD, and the others showed either ERS or negligible change.

## Discussion

In accord with the aims and hypotheses of this study, left and right μ components were identified across perception and production tasks. 85 and 75% of participants submitted components with ~10% unexplained RV in left and right μ components, respectively. This proportion of useable μ components is similar to that found in other studies (e.g., Nystrom, 2008; Bowers et al., 2013), though the proportion of unexplained RV is slightly higher, possibly due to the inclusion of motor tasks. Bilateral localization of μ rhythm source maxima to the precentral gyrus with activity spreading across the premotor and sensorimotor cortices is consistent with accepted sources of the rhythm (Pineda, 2005; Hari, 2006) and important roles in speech perception/production (Skipper et al., 2007; Sato et al., 2009; Callan et al., 2010; Houde and Nagarajan, 2011; Tourville and Guenther, 2011). As these cortical sites are known to play important roles in SMI for both speech perception and production and μ rhythms are comprised of frequency bands that are sensitive to the demands of speech processing, this finding supports the subsequent examination of real-time activity within these clusters for better understanding the temporal dynamics of activity in the dorsal speech stream.

### Time-frequency analyses in perception conditions

Similar to previous investigations (Makeig et al., 2004; Graimann and Pfurtscheller, 2006; Hari, 2006), anticipation of a button-press response in the Pasn condition yielded low-level increases from baseline in bilateral μ-ERD that gained slightly in strength with temporal proximity to the response. Discrimination conditions herein (Qdis and Ndis) also employed button-press responses, hence controlling for this effect in the statistical analysis. The Qdis and Ndis conditions produced similar highly accurate syllable discriminations and response reaction latencies such that differences between μ ERS/ERD in these conditions are attributable to the presence or absence of noise. Both conditions produced similar bilateral patterns of μ ERS/ERD that were generally stronger in the left hemisphere than the right, supporting left hemisphere dominance for SMI in speech perception (Hickok et al., 2011).

#### μ-alpha

Activity in the alpha band was characterized initially by ERS occurring prior to stimulus onset. ERS gradually gave way to ERD (Figure 5), with suppression first in low alpha (i.e., 8–10 Hz) and then high alpha (11–13 Hz). Alpha ERS was stronger and the transition occurred later in the Ndis condition than in the Qdis condition. Alpha rhythms are found globally across the cortex and their power can vary with numerous cognitive states and processes (Klimesch, 2012). Therefore, it is necessary to interpret alpha ERS/ERD relative to the tasks that induced them. Enhanced alpha (i.e., ERS) often is associated with cognitive load in working memory and attention tasks (Leiberg et al., 2006; Jensen et al., 2007; Haegens et al., 2010). It is thought to be an index of cortical inhibition of sensory information irrelevant to a given task, functioning to help sharpen attention to relevant information (Klimesch, 2012; Wilsch et al., 2014). In speech perception, this type of “active sensing” has been described in phenomena such as in the “cocktail party” effect, where specific attention to relevant speech cues helps filter similar competing background speech (Schroeder et al., 2010; Zion Golumbic et al., 2012).

Weisz et al. (2011) provide compelling evidence for an independently generated auditory alpha that is responsive to speech perception. Parsimonious with notions of increased cognitive load and consistent with the current findings, signal degradation of speech by noise vocoding has also been shown to enhance alpha activity (Obleser and Weisz, 2012). The observed differences in early alpha ERS between the Qdis and Ndis conditions support these notions. On the other hand, in speech perception tasks, alpha ERD has been found while evaluating speech (Shahin et al., 2009). Late occurring posterior alpha ERD has been related to increased speech intelligibility (Obleser and Weisz, 2012). Both of these findings are consistent with the notion of alpha ERD during accurate performance of perceptual and memory tasks (Klimesch et al., 2006). In addition, μ-alpha is suppressed in auditory speech perception tasks (Cuellar et al., 2012; Pineda et al., 2013). Hence, the current findings of late alpha ERD suggest that following stimulus offset, the two syllables were being evaluated by participants in the decision-making process.

#### μ-beta

In both discrimination conditions, significant beta ERD (relative to Pasn) was found across the time course of trials, prior to, during, and after acoustic stimulation. Beta ERD spread from narrow (17–19 Hz) to wide (15–30 Hz) beta bands while gaining in strength in both discrimination conditions. However, beta ERD occurred earlier in the Qdis than the Ndis condition. Bowers et al. (2013) previously showed that early beta ERD occurred when discriminating speech but not tones. They suggested that in speech perception tasks, early beta ERD also can be explained as a function of predictive coding. That is, internal models are posited to be generated in motor regions that are delivered to higher order auditory regions (i.e., superior temporal sulcus) to help constrain analysis and functionally improve speech discrimination accuracy (Callan et al., 2010; Bowers et al., 2013). These findings are also consistent with those of Mottonen et al. (2013), in that degraded conditions do no appear necessary to induce motor activity in speech discrimination. These models are thought to be available because of the considerable experience of humans generating the movements that produce these sounds. In addition, this predictive coding may have been fine tuned within the experiment. That is, requiring participants to accurately discriminate syllables 160 times (80 per condition) may have elicited anticipatory attention to speech processing.

#### μ-alpha and beta in discrimination conditions

The patterns of alpha and beta μ ERS/ERD found in quiet and noisy accurate speech discrimination need to be considered in combination. While similar patterns were observed in Qdis and Ndis conditions, stronger early alpha ERS was observed in the Ndis condition, which is consistent with the requirement of discriminating in noise. That is, it is speculated that the inhibitory mechanism was stronger when background noise was present. Conversely, early beta suppression appeared to be stronger in the Qdis than the Ndis condition. Though it is likely that internal models were generated in both conditions since they both used speech and were discriminated accurately (Bowers et al., 2013), it appears that in this study, the strong alpha ERS may have dominated the μ rhythm, extended into the low beta frequencies in the Ndis condition, and perhaps negated some early beta ERD. Together, these data suggest that alpha ERS and beta ERD within the sensorimotor μ rhythms work in unison, co-operating to functionally support accurate speech discrimination. This is further evidence that examination of the μ-rhythm provides a rich, time-sensitive, and relatively unique view of SMI in speech discrimination from an oscillatory perspective.

#### Dorsal stream motor activity in speech perception

The source location of μ clusters and their alpha and beta ERS/ERD suggest that they provide important information regarding sensorimotor dorsal stream activity in speech perception. The current findings suggest that beta activity may provide a measure of predictive coding via internal models (Bowers et al., 2013) generated in the PMC (Skipper et al., 2007; Houde and Nagarajan, 2011; Tourville and Guenther, 2011; Rauschecker, 2012). Tamura et al. (2012) investigated μ rhythm activity in various speech tasks, including covert production and production under different types of auditory feedback. They found differential activity within the alpha band and concluded that the μ-alpha was an index of auditory monitoring for speech. In line with this notion, it is speculated that μ-alpha might index sensory feedback into the PMC. Thus, stronger alpha ERS in the Ndis condition was observed, possibly due to a stronger inhibition of auditory feedback to the PMC when speech was presented in background noise. Furthermore, in the time period following stimulus offset and prior to the button press response, it seems likely that the two syllables were held in working memory, while being compared and covertly replayed during the decision-making process. These processes may require the generation of internal speech models and the disinhibition of feedback to the PMC, which would support the current findings of alpha and beta ERD in this time period within trials.

### Time-frequency analyses in production conditions

The covert (Img) and overt (SylP and WorP) production conditions yielded similar general patterns of alpha/beta ERD relative to baseline across trials. However, both alpha and beta ERD were significantly stronger in the overt production conditions than the Img condition, with significant differences in ERD following the cue to speak in both conditions. Across production conditions, there appeared to be little difference between right and left μ ERD. This is consistent with others that have found movement-induced bilateral decreases in beta suppression across the sensorimotor cortex (e.g., Salmelin and Hari, 1994; Pfurtscheller et al., 1996; Stančák and Pfurtscheller, 1996; Leocani et al., 1997, 2001; Alegre et al., 2003; Rau et al., 2003; Bai et al., 2005; Doyle et al., 2005; Erbil and Ungan, 2007). No differences between SylP and WorP conditions were observed. As expected, ERSP time-frequency analysis of perilabial EMG activity showed little activity in the Img condition, confirming that participants did not articulate the target syllables. In the SylP and WorP conditions, EMG activity following the “go” cue to speak was characterized predominantly by strong ERS beginning ~300 ms in both conditions. This time lag from the “go” cue is consistent with a normal movement reaction time. Hence, μ-alpha and μ-beta ERD showed temporal alignment to lip muscle movements.

#### μ-alpha during production

μ-alpha ERD in speech production is again interpreted as an index of feedback to the PMC while speech is being produced. By only measuring activity in the sensorimotor μ, it is not possible to differentiate between auditory and somatosensory feedback. μ-alpha suppression is traditionally localized to the somatosensory cortex and considered to reflect somatosensory activity (Hari, 2006). However, in light of recent findings in perception (Cuellar et al., 2012; Tamura et al., 2012; Pineda et al., 2013), there is mounting evidence that it also may reflect auditory feedback. In speech production, this makes sense considering how both auditory and somatosensory integration regions provide feedback to the PMC during speech production. Furthermore, the feedback from the auditory system and somatosensory system are generally consistent during speech production such that, barring perturbation to either modality, SFC models allow for them to often be considered unitarily (Houde and Nagarajan, 2011).

#### μ-beta during production

During overt production (SylP and WorP), beta μ-ERD is easily explained as a consequence of motor activity. Sensorimotor beta power has been ubiquitously found to suppress to motor activity from effectors including the fingers (Gaetz et al., 2010), wrist (Alegre et al., 2003), shoulder (Stančák et al., 2000), foot (Pfurtscheller and Lopes Da Silva, 1999), and tongue (Crone et al., 1998). However, if SFC models are applied, beta ERD can be cautiously interpreted as an index of PMC activity in the generation of feedforward control to motor effectors and forward internal models (efference copies) to the feedback loop. This interpretation is supported in a recent review (Engel and Fries, 2010; Kilavik et al., 2013), suggesting the difficulty in determining a clear functional role of sensorimotor beta suppression during movement, but that it may reflect sensory and cognitive aspects (e.g., forward modeling) in addition to pure motor processes. That said, one limitation of the current interpretation is the inability of beta ERD to distinguish between motor activity in feedforward (i.e., muscle movements) and feedback (i.e., internal modeling) mechanisms.

#### Covert speech production

The Img (covert production) produced significantly weaker alpha/beta ERD than the overt production conditions. This condition was incorporated into the design as previous work examining motor imagery and covert speech production had shown patterns of μ suppression and sensorimotor activity similar to overt productions (Pfurtscheller and Lopes Da Silva, 1999; Neuper et al., 2006). However, there is also evidence that responses in these covert conditions have been weaker than in actual overt productions (Neuper et al., 2006). In a recent study, Holler et al. (2013) investigated μ activity to real and imagined hand movements and showed that only 11 of 18 participants produced differences in μ-alpha/beta power when imagining hand movements. Of these 11, two showed μ enhancement rather than the suppression that was shown in the real movement conditions, suggesting variable responses to covert production tasks. The results in the current Img condition showed similar variability, perhaps contraindicating future use of covert production over a large number of repeated trials. This was the only condition in the experiment that required no overt response (either button-press or speech production) and hence, it was impossible to monitor the extent of covert syllable productions that was asked of participants 80 times in this condition.

#### Early μ-ERD in overt production

Significantly strong μ-ERD (relative to Img) was found as speech was being produced. Weaker μ-ERD was observed in SylP and WorP conditions prior to production and even before the cue to speak. This time period coincided with the preparation of the speech network, during which similar oscillatory activity has been reported (Gehrig et al., 2012; Herman et al., 2013). μ-ERD during this time period prior to production was weaker than expected, especially in light of the findings in the speech perception tasks, and predictions from SFC models (Houde and Nagarajan, 2011). This reduced neural ERD was most likely due to the influence of EMG on overall EEG variance.

### The utility of ICA in speech perception and production

μ components were successfully identified from band-pass filtered concatenated EEG data from perception and production conditions. Though the unexplained RV of the average μ ECD was slightly higher than has been found in other studies (e.g., Bowers et al., 2013), the combination of ECD/sLORETA CSD techniques produced a reliable and valid estimate of μ sources within the standard head model that was applied to all ICA data.

In the perception conditions, time-frequency analyses revealed differential contributions from alpha and beta bands of the μ rhythm that contributed to accurate syllable discrimination. μ-alpha/beta ERD was also revealed in speech production synchronized to muscle activity. This pattern of activity had not been described previously and can be interpreted as being consistent with “normal” sensorimotor control in speech production. Future investigations involving auditory or somatosensory speech perturbations (e.g., Bauer et al., 2006; Reilly and Dougherty, 2013) might be expected to reveal differences in alpha/beta ERD in speech production. Similarly, different relative patterns of μ ERS/ERD might be observed in clinical populations with compromised sensorimotor control such as in stuttering (Max et al., 2003; Loucks and De Nil, 2006; Watkins et al., 2008; Hickok et al., 2011; Cai et al., 2014; Connally et al., 2014).

In addition to the positive findings, there was also evidence of drawbacks to using EEG/ICA in production tasks. It was clear that ICA adequately separated neural from non-neural (e.g., myogenic) activity. Had this not been successfully accomplished, μ-ERD/ERS during production would likely have been overwhelmed by the EMG activity. However, it also appears that overall spectral power in μ components was reduced in the production tasks due to a greater proportion of the overall EEG variance that had to be accounted for by EMG activity. Considering motor requirements, strongest μ-ERD (especially beta) would have been expected in production conditions. However, even when at their strongest, spectral powers during production did not exceed those in perception. In addition, only weak μ-ERD was noted in the time period prior to overt production, which was expected to be stronger as the speech networks prepared to articulate. Together, these findings indicate that overall spectral power in production conditions was attenuated. As such, though interesting general patterns of μ-ERD were revealed in speech production, they should be interpreted with caution with respect to their sensitivity and without making reference to function in conditions without motor requirements.

Another limitation in the current methods was the inability to observe μ activity following speech production. Though production targets (i.e., syllables and words) were produced within the time course of trials, EMG activity (e.g., lip movement) persisted past production, such that the epoch length that did not allow for the measurement of beta rebound (i.e., ERS), which is commonly observed following termination of a movement (Kilavik et al., 2013).

### Conclusions and future directions

ICA successfully identified μ components in speech perception and production. Time-frequency analyses using ERSP showed real-time changes in alpha/beta power that provided indicators of PMC/sensorimotor contributions to speech-based dorsal stream activity. Localization of μ clusters and ERSP activity in perception and production are in agreement with Rauschecker's (2011) observation that, based on connections to the inferior parietal lobe and posterior auditory cortex, the PMC provides “optimal state estimation” for speech.

Sensitivity of the findings was somewhat reduced in production conditions, most likely due to concomitant myogenic activity. Further applications in speech production might consider additional filtering techniques in addition to ICA. Exquisite temporal resolution combined with economy and availability warrant further use of ICA particularly to understand speech processing in normal and clinical populations. While measuring the temporal dynamics of the μ-rhythm provide rich information about sensorimotor processing, future ICA studies may also investigate multiple components within the speech processing network in addition to measuring connectivity (i.e., coherence) between components.

### Conflict of interest statement

The authors declare that the research was conducted in the absence of any commercial or financial relationships that could be construed as a potential conflict of interest.
